# Safety, efficacy and timing of antithrombotic therapy in emergency stenting of acute stroke patients with tandem lesions, German multicenter data-analysis

**DOI:** 10.3389/fneur.2025.1554691

**Published:** 2025-04-02

**Authors:** Fee Keil, Simon Stahn, Ferdinand O. Bohmann, Patrick Samp, Leonhard Mann, Lukas Bersch, Waltraud Pfeilschifter, Felix Bode, Marios-Nikos Psychogios, Jan-Hendrik Schaefer, Christian Grefkes, Elke Hattingen, Joachim Berkefeld, Christophe T. Arendt, German Stroke Registry – Endovascular Treatment (GSR-ET) Investigators, Joachim Röther, Bernd Eckert, Michael Braun, Gerhard F. Hamann, Eberhard Siebert, Christian H Nolte, Martina Petersen, Lars Krause, Tobias Boeckh-Behrens, Silke Wunderlich, Maximilian Schell, Jens Fiehler, Götz Thomalla, Fabian Flottmann, Anna Alegiani, Franziska Dorn, Gabor Petzold, Jan Hendrik Schäfer, Fee Keil, Hanna Zimmermann, Steffen Tiedt, Lars Kellert, Ulrike Ernemann, Sven Poli, Arno Reich, Omid Nikoubashman, Ilko L Maier, Marielle S Ernst, Mario Abruscato, Sven Thonke, Arman Gregor, Evdokia Evangelidou, Peter Schellinger, Jan Borggrefe

**Affiliations:** ^1^Institute of Neuroradiology, Goethe University Frankfurt, University Hospital, Frankfurt am Main, Germany; ^2^Department of Radiology, Hospital Nordwest, Frankfurt am Main, Germany; ^3^Department of Neurology, Goethe University Frankfurt, University Hospital, Frankfurt am Main, Germany; ^4^Department of Neurology, Hospital Lüneburg, Lüneburg, Germany; ^5^Department of Vascular Neurology, Center of Neurology, University Hospital Bonn, Bonn, Germany; ^6^Department of Neuroradiology, University Hospital Basel, Basel, Switzerland

**Keywords:** platelet aggregation inhibitors, stents, thrombectomy, thrombotic stroke, carotid artery thrombosis, carotid artery stenosis, tandem lesions, tandem occlusion

## Abstract

**Background:**

Antithrombotic therapy of acute stroke patients with tandem lesions and emergent carotid artery stenting (CAS) is still a matter of controversial debates. The lack of evidence from dedicated studies favors a variety of clinical practices. The aim of this study was to use German Stroke Registry (GSR) data of selected high-volume centers to analyze the spectrum of antithrombotic regimens and their influence on complication rates and clinical outcome.

**Methods:**

We analyzed the GSR-subgroup of patients with tandem lesions treated with a combination of thrombectomy and carotid artery stenting between 2015 and 2020 in experienced stroke centers which included all consecutive cases, and which were willing to share clinical records in addition to registry data. Statistical analyses of kind and onset of CAS-specific antiplatelet therapy (APT) were used to determine the influence of dual APT (DAPT) on clinical outcome and stent-related complications.

**Results:**

In total, 223 patients with tandem lesions treated by stenting and thrombectomy were included. In the periinterventional phase 68 patients (30.5%) had an aggressive DAPT with double antiplatelets (DAPT; 23.7%) given via gastral tube or with GPIIb-IIIa inhibitors intravenously as bridging (13.9%). In the post-interventional phase, the rate of DAPT increased from 38.6% on day one to 65% from day two. Already on day one, the effect of DAPT on the rate of good clinical outcome mRS (modified Rankin Scale) 0–2 at 90 days (*n* = 86/223; 38.5%) was significant (*p* < 0.007), and compared with other APT regimens early continuous DAPT from the first postinterventional day increased the odds up to 79.4% (*n* = 27/34). DAPT during hospitalization showed no increased risk of symptomatic hemorrhage, while post-hospital use reduced stent occlusion (*p* = 0.016).

**Conclusion:**

Only a minority of the examined GSR patients with tandem lesions had an effective APT during the periinterventional phase up to day 1. Early postinterventional DAPT significantly increased the rate of good clinical outcome and reduced the rate of occlusive stent thrombosis without increasing risks of symptomatic hemorrhage. The apparent lack of standards and moderate rates of good clinical outcomes shows room for improvement and the necessity of further studies.

## 1 Introduction

Multiple randomized controlled trials (RCT) have demonstrated the efficacy of endovascular treatment (ET) of patients with acute ischemic stroke caused by large vessel occlusion (LVO). A benefit from interventional recanalization is also proven for the subgroup of patients with tandem lesions -extracranial carotid stenosis and intracranial embolic LVO (1). Stroke patients with tandem lesions are most frequently treated by emergent intracranial thrombectomy and carotid stenting (CAS). The endovascular procedure is more complex than a simple thrombectomy and guideline recommendations for CAS demand an effective double antiplatelet therapy (DAPT) for prevention of stent thrombosis (2). The question is whether this is also true for emergent CAS during acute stroke interventions. Preinterventional thrombolysis and thrombectomy are associated with risks of intracranial hemorrhage (ICH) which may be increased by aggressive DAPT. Between neurointerventional centers it was especially discussed whether effective DAPT should be established before emergent stent deployment or whether it is better to start with a single antiplatelet, followed by DAPT after exclusion of hemorrhage at the day after the intervention or even later. Up to date this question has not yet been addressed by dedicated RCT.

Due to inconsistent recanalization results the option of balloon angioplasty without stenting is not widely accepted (3). Studies identifying stent thrombosis as a significant risk factor for poor clinical outcome support the necessity of antiplatelets (4). There is no final evidence concerning kind and timing of emergent APT: Data from multicentric studies suggest that a state after thrombolysis and an effective DAPT are not associated with an increased risk of symptomatic hemorrhage (5–8). Concerns that emergent DAPT or the use of GP IIb/IIIa-inhibitors may increase the risk of bleeding led to the implementation of dedicated RCT (9).

Facing this background, it was the purpose of this subgroup analysis from German Stroke Registry (GSR)- data to describe practices of emergent antithrombotic therapy in patients with tandem lesions. Statistical evaluation aimed at answering the question whether peri- and postinterventional antiplatelet regimens are significantly associated with CAS-related complications like stent occlusion or symptomatic ICH (sICH). We also sought to determine the influence of kind and onset of APT on clinical outcomes at discharge and after 3 months.

## 2 Patients and methods

The patients analyzed in this study were derived from the German Stroke Registry (GSR), an ongoing prospective, multi-center registry with multiple participating sites in Germany including all consecutive patients who suffer from acute ischemic stroke with LVO treated by ET (GSR-ET; ClinicalTrials.gov, identifier: NCT03356392). Study design and major findings have already been published (10). Unlike typical registry-based studies, our data were meticulously gathered and validated through comprehensive on-site investigations and detailed review of patient records. This rigorous approach ensures a higher level of accuracy and reliability, setting our study apart and providing robust insights into current practices.

### 2.1 Inclusion criteria

**Figure 1:**
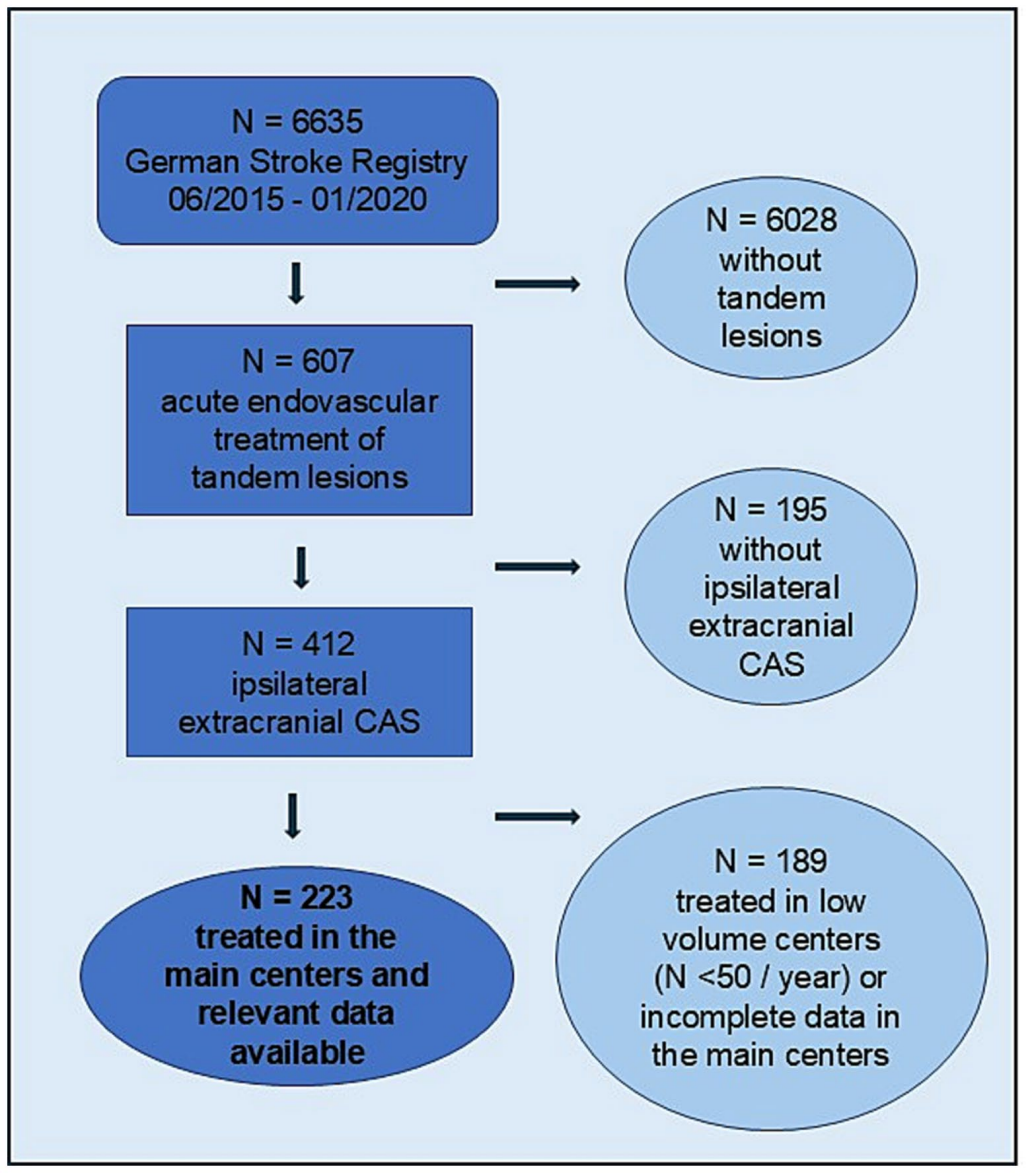
Flowchart of patient selection for the study. Flowchart depicting the patient selection process, including screening, application of inclusion and exclusion criteria, and final enrollment in the study. CAS, carotid artery stenting.

For this analysis, we included patients with tandem lesions who underwent thrombectomy of an intracranial LVO and concomitant stenting of a relevant ipsilateral extracranial carotid artery pathology (stenosis >70% according to NASCET, North American Symptomatic Carotid Endarterectomy). Patients were older than 18 years and enrolled in the registry between June 2015 and January 2020. We included only data of patients treated in centers that enrolled at least 50 thrombectomy patients per year and had entered the registry with >90% data completeness. According to a lack of detailed registry data the included centers were furthermore willing to share further clinical and imaging records. At the time of the data analysis, 12 centers fulfilled these conditions. Figure 1 shows the data selection criteria.

### 2.2 Exclusion criteria

Patients who did not undergo CAS or patients with occlusions on the basis of intracranial atherosclerosis were excluded from this evaluation.

The recorded data from the GSR database were pre-analyzed and compared and supplemented with clinical records of medical treatment, intervention laboratory and imaging data.

Intravenous thrombolysis therapy (IVT) using recombinant tissue Plasminogen Activator (body weight-adapted dose: 0.9 mg/ kg; 10% as a bolus, 90% as continuous infusion over 1 h) was applied prior to intervention as appropriate according to national and international guidelines if there were no contraindications. Depending on the respective center, the heterogenous local standard of care regarding the use of APT was documented. The evaluation was focused on pre-, peri-, and post-interventional APT. Type and dosage of thrombolytics, anticoagulants and especially antiplatelet agents were recorded during the first 48 h and up to discharge. Continuous effective APT was defined as DAPT from day 1 up to discharge. Aggressive DAPT was defined as DAPT with periinterventional start given via gastral tube or with GPIIb-IIIa inhibitors i.v. as bridging and oral continuation up to discharge.

Neurological outcomes and disability were assessed by the modified Rankin Scale (mRS). Imaging data from postinterventional Doppler-, CT- or MRI-examinations were used for the detection of stent occlusion, ICH or infarction. SICH was defined according to the Heidelberg criteria (11).

Clinical outcome was assessed by neurologists at discharge and after 90 days. The mRS was used for description of clinical outcomes and disability.

Additional data were used for patient characterization and for the statistical analysis of potential confounders different from antithrombotic therapy such as sex, age and baseline National Institutes of Health Stroke Scale (NIHSS), occlusion site, and Alberta Stroke Program Early CT Score (ASPECTS). Angiographic success of intracranial recanalization was assessed due to the mTICI scale (12). CAS associated complications were detected by Doppler ultrasound and/or CT angiography for occlusive stent thrombosis and native cCT or cMRI for ICH. In case of ICH, worsening of symptoms upon neurological examination defined sICH provided that NIHSS decreased by ≥4 points.

Interventional therapy with antegrade (CAS first) or retrograde approach (thrombectomy first) and implantation of a self-expanding carotid artery stent was performed by choice of the participating centers.

### 2.3 Endpoints

Primary endpoint of this study was the rate of CAS associated complications: stent occlusion and sICH.

Secondary endpoint was the rate of poor clinical outcomes mRS > 2 at discharge and after 3 months.

### 2.4 Statistical analysis

Standard descriptive statistics are reported as mean and median value for continuous and ordinal variables. Numbers and percentage were used for categorical variables. For between-group comparisons of categorical variables, χ^2^-tests or Fisher exact tests were used, as appropriate.

Univariate and multivariate binary logistic regression analyses were applied to identify predictors for stent occlusion, sICH and good clinical outcome mRS 0–2 at discharge and after 90 days. A special focus of the analysis was whether kind and timing of APT was associated with clinical outcome. Besides anticoagulation and antiplatelet medication, particular variables, which are known predictors of outcome after stroke thrombectomy, were included: age, pre-existing conditions, NIHSS on admission, ASPECTS as continuous independent variables, and concomitant IVT, successful recanalization (defined by modified Thrombolysis in Cerebral Infarction (mTICI) grades 2b-3) as dichotomous independent variables.

The resulting relative risk reduction, Odds Ratio and *p* values are presented. Analysis was exploratory, and *p*-values <0.05 were considered statistically significant. For the primary endpoint, we corrected the *p*-value with Bonferroni for multiple tests to <0.025. All tests were two-sided. The statistical analysis was performed using SPSS (Version 29.0; IBM, Armonk, New York).

## 3 Results

### 3.1 Patient data

We included registry data and records of 223 acute stroke patients from 12 centers who had interventional treatment of tandem lesions with a combination of intracranial thrombectomy and emergent CAS between 06/2015 and 01/2020.

Sixty eight patients (30.5%) were female and 155 (69.5%) were male with a mean age of 71 years (range: 38–93) (Table 1).

**Table 1:** Patient characteristics.

	n	%
Thrombectomy patients with tandem lesions and emergent CAS	223	100
Age (mean: 71.0 years; range: 38–93 years)		
38–59 years	37	16.6
60–69 years	59	26.5
70–79 years	66	29.6
≥80 years	61	27.4
Gender		
Females	68	30.5
Males	155	69.5
Risk factors		
Arterial blood pressure on admission >140 mmHg systolic	131	58.7
Arterial Hypertension	162	72.6
Diabetes	65	29.1
Dyslipidemia	72	23.2
Atrial fibrillation	32	14.3
Hospital admission		
Direct admission	109	48.9
Transfer from another hospital	114	51.1
NIHSS (median: 13; range: 1–29)	(221)	
NIHSS <6	29	13.1
NIHSS 6–15	114	51.6
NIHSS >16	78	35.3
ASPECTS (median:9; range: 2–10)	(215)	
ASPECTS <3	1	0,5
ASPECTS 3–5	16	7.4
ASPECTS 6–8	87	40.5
ASPECTS 9–10	111	51.6
Missing data	8	3.6
Antithrombotic medication before admission		
Antiplatelets (ASA or/and Clopidogrel)	69	30.9
Anticoagulants	19	8.5

**Figure 2:**
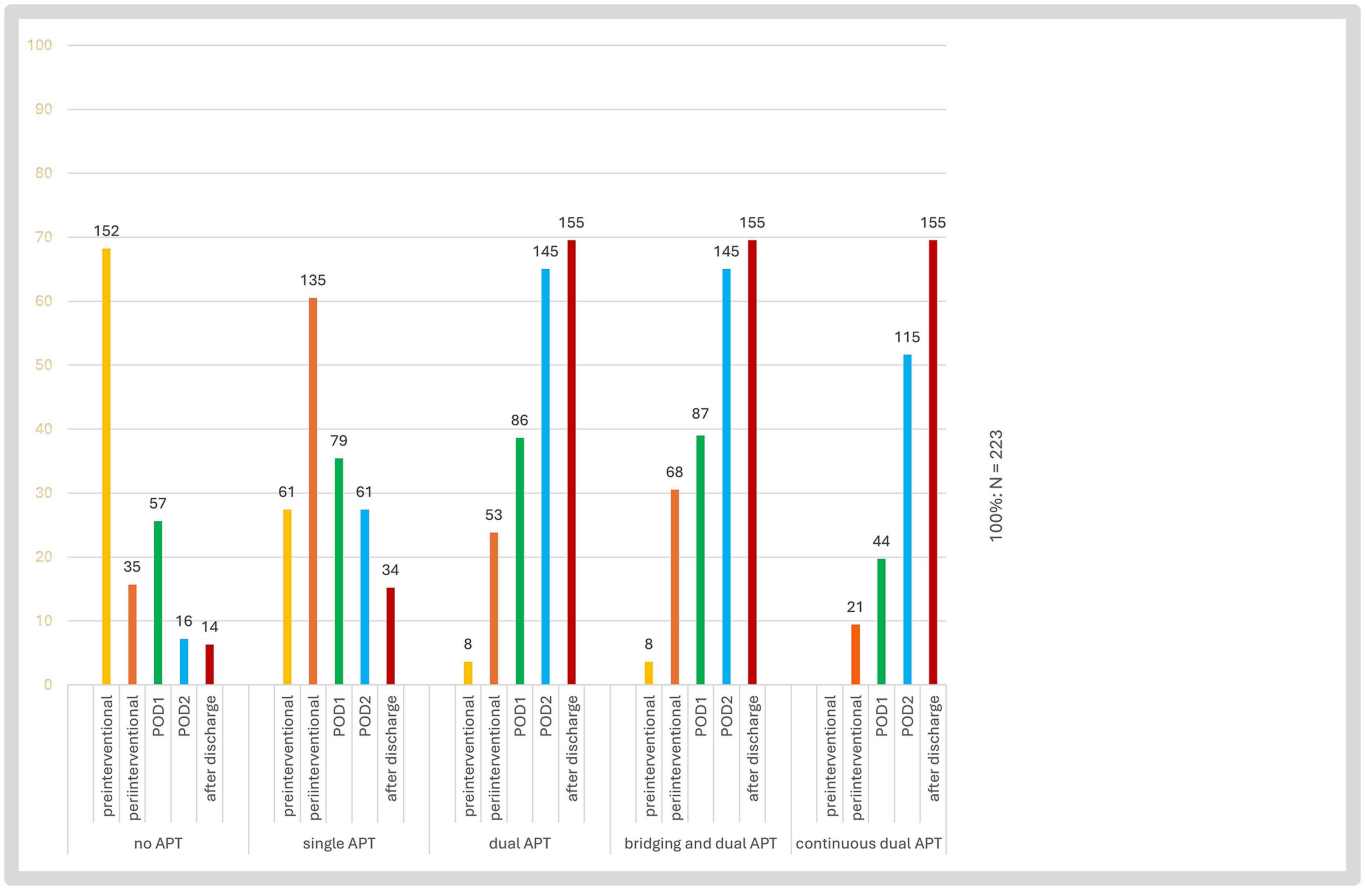
Antiplatelet therapy regimens in acute endovascular treatment with tandem lesions. This bar chart illustrates the distribution of different antiplatelet therapy (APT) regimens over time in 223 stroke patients undergoing acute ipsilateral extracranial carotid artery stenting for tandem lesions. The y-axis represents the percentage of patients, while the absolute number of patients for each category is displayed above each bar. The x-axis categorizes different APT strategies. The color-coded bars indicate therapy administration at different time points. POD1, post-operative day 1; POD2, post-operative day 2.

**Figure 3:**
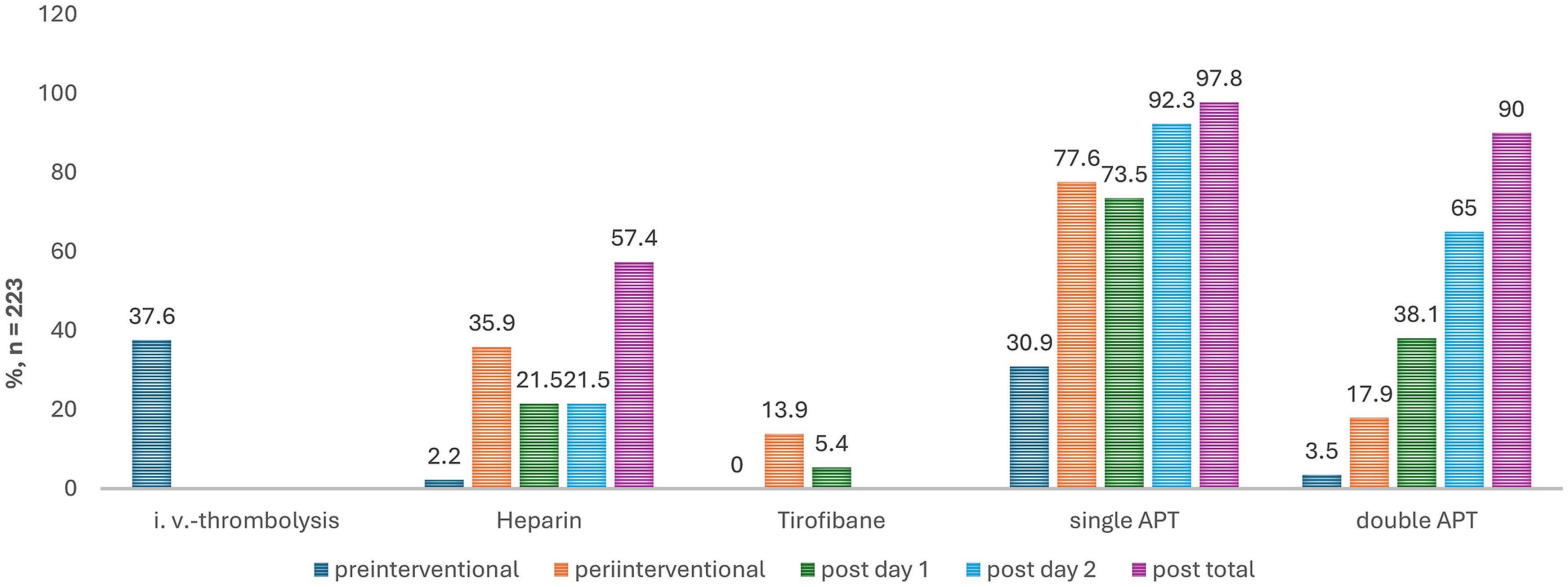
Type of anticoagulation over time in stroke patients with tandem lesions undergoing thrombectomy and carotid artery stenting. This bar chart illustrates the distribution of various types of anticoagulation therapy administered to 223 stroke patients undergoing acute ipsilateral extracranial carotid artery stenting for tandem lesions. The y-axis represents the percentage of patients, while the absolute number of patients for each category is indicated above each bar. The x-axis categorizes different types of blood thinners used. Each bar is color-coded to reflect different timing of administration, enhancing the visual differentiation of immediate versus later treatment. POD1, post-operative day 1, POD2, post-operative day 2.

Figures 2, 3 show kind and timing of the pre-, peri- and postinterventional antithrombotic therapy with additional medication. 36.8% of the patients had pretherapeutic APT or anticoagulation; 62.3% out of 223 patients pre-interventional i. v.-thrombolysis. During the periinterventional phase 60.5% had a single and 23.8% a DAPT. Bridging with i. v. Tirofiban was recorded in 31 patients, combined with a single antiplatelet in 11 cases and additional Heparin in another 16 cases and with dual antiplatelets in 5 cases. Regardless of the use of Tirofiban, Heparin was given in 35.9% of cases.

During the postinterventional phase the rate of DAPT increased from 23.8 periinterventional to 38.6% at day 1 up to 65.0% at day 2.

In the majority of the 223 patients (76.6%; n: 171) a combination of Acetylsalicylic Acid (ASA) and Clopidogrel was administered for DAPT. Ticagrelor was used instead of Clopidogrel in 30 cases (13.5%).

### 3.2 Univariate binary logistic regression analysis and multivariate regression analysis

**Figure 4:**
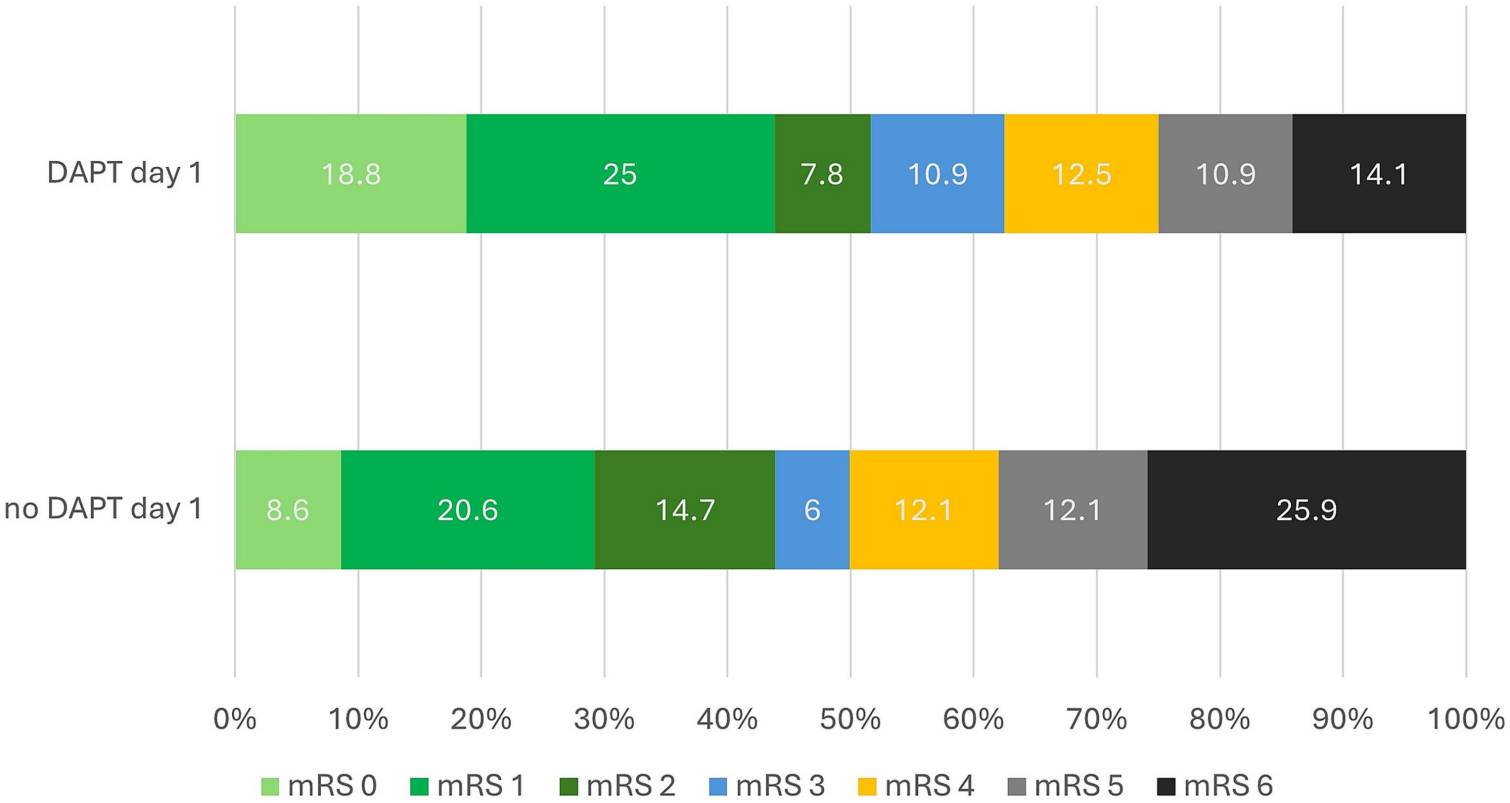
90-Day clinical outcomes after thrombectomy and carotid artery stenting with versus without dual antiplatelet therapy on post-operative day 1. This bar chart compares the 90-day outcomes of stroke patients using the modified Rankin Scale (mRS), contrasting those who received dual antiplatelet therapy (on the first post-operative day with those who did not). The y-axis shows the percentage of patients by mRS score from 0 to 6, with bars divided into upper (DAPT) and lower (no DAPT) sections. Bars are color-coded to visually separate good outcomes (mRS 0–2) from poorer outcomes (mRS 3–6), with a diagonal line delineating the threshold between mRS 2 and 3 to highlight this transition. Percentages are displayed within each bar segment. Multivariate analysis revealed a significant difference in good outcomes: 51.6% in the DAPT group versus 43.9% in the non-DAPT group (*p* = 0.034).

Univariate regression analysis identified a significant risk reduction for DAPT from Day 2 during the hospitalization (*p* = 0.033). Even the risk of any ICH was not increased by double antiplatelets. ICH with DAPT 23.2% (44/190) without 42.4% (14/33). The risk of stent thrombosis with carotid artery occlusion was significantly reduced by DAPT in the post hospitalization period (*p* = 0.016) but not by single APT. Continuous DAPT from day 1 to day 90 was associated with a significantly higher rate of favorable clinical outcome at day 90 (OR 6.5; 2.63–15.80; *p* < 0.001), these patients had the best chance of a favorable clinical outcome at 90 days, with a rate of 79.4% (27/34) instead of 37.4% (55/147) with none, late or discontinuous DAPT. This effect is shown in Figure 4.

A significant effect of DAPT or Tirofiban bridging could not yet be detected in the periinterventional phase (OR 1.2; 0.64–2.23; *p* = 0.581) but already on day 1 (OR 2.3; 1.24–4.26; *p* = 0.008).

Multivariate regression analysis confirmed these results and identified DAPT from day 1 as significant predictor for good outcome (OR 2.3; 1.06–4.81; *p* = 0.034).

Neither pre- nor post-interventional deviations of coagulation parameters like PTT, INR and platelet count showed relevant influence on the risk of bleeding or stent thrombosis. Fourteen patients had pre-medication with Warfarin or NOAKs, 12 of these patients due to atrial fibrillation, 5 of them had additionally received IVT pre-intervention. Only one of these 14 patients developed symptomatic bleeding, which was not significant. Peri- and postinterventional, 31 patients received Tirofiban, 12 patients even on the first postintervention day; here, too, there was no significant increase of the risk of bleeding. Periinterventional Heparin had also no significant effect on the outcome. Apart from antithrombotic therapy significant risk factors for poor outcome were age > 60 (*p* < 0.001), severe stroke NIHSS >15 (*p* < 0.001), AF (*p* = 0.042) and large infarcts on pre-interventional imaging with ASPECTS <6 (*p* = 0.040).

Risk factors for poor outcomes are shown in Table 2.

**Table 2:** Factors influencing poor clinical outcome (181/223 datasets after 90 days).

mRS ≤ 2 after 90 days *n* = 82/181						
*n* = 181	n	%	mRS ≤ 2	%AR	RR	p*
Age > 60	**143**	**79.0**	**55**	**38.5**	**0.54**	**<0.001**
Age < 60	38	21.0	27	71.1	1.85	
*n* = 168						
Pathologic coagulation values	25	14,9	9	36.0	0.76	0.285
Normal coagulation values	143	85.1	68	47.6	1.32	
*n* = 181						
Atrial fibrillation	**26**	**14.4**	**7**	**26.9**	**0.56**	**0.042**
No atrial fibrillation	155	85.6	75	48.4	1.80	
Hypertension	131	72.4	55	42.0	0.78	0.146
No hypertention	50	27.6	27	54.0	1.29	
*n* = 179						
NIHSS >15	61	34.1	16	26.2	0.48	**<0.001**
NIHSS ≤15	118	65.9	65	55.1	2.10	
*n* = 173						
ASPECTS ≤5	**12**	**6.9**	**2**	**16.7**	**0.35**	**0.040**
ASPECTS >5	161	93.1	76	47.2	2.83	
Periinterventional antiplatelet therapy *n* = 181						
Tirofiban	30	16.6	18	60.0	1.42	0.077
No Tirofiban	151	83.4	64	42.4	0.71	0.077
Intravenous thrombolysis	113	62.4	53	46.9	1.10	0.578
No Intravenous thrombolysis	68	37.6	29	42.6	0.91	0.578
Dual antiplatelet therapy	58	32.0	28	48.3	1.10	0.581
No dual antiplatelet therapy	123	68.0	54	43.9	0.91	0.581
Mono antiplatelet therapy	98	54.1	46	46.9	1.97	0.872
No antiplatelet therapy	25	13.8	8	32.0	0.66	0.17

*p-values calculated with χ^2^-test. Bold values indicate the significant findings.

### 3.3 Angiographic and clinical results

A good technical recanalization result with mTICI 2b-3 was achieved in 203 (91%) of 223 patients.

Angiographic results and outcome are shown in Table 3.

**Table 3:** Vascular and clinical outcome.

Outcome	n	%
Successful recanalization mTICI 2b-3	203	91
Good clinical outcome mRS 0–2		
At discharge	68	30.5
After 90 days	82	36.8
Mortality in hospital	22	9.9
Mortality after 90 days	43	19.3
Complications		
Any intracranial hemorrhage	58	26.0
Symptomatic intracranial hemorrhage	15	6.7
Stent thrombosis	24	10.8
Stent occlusion	18	8.1

Sixty eight (30.5%) of all patients were discharged with a good outcome (mRS ≤2). Of the five patients in whom the occluded vessel could not be reopened (TICI 0), three died (mRS of 6), and the other two patients were discharged with an mRS of 5. In 15 patients only a partial reperfusion with <50% (TICI 2a) could be achieved, of which 20% could be discharged with an mRS ≤2.

The rate of good clinical outcomes improved from 30.5% at discharge to 36.8% at 90 days. The in-hospital mortality rate was 9.9 and 19.3% at 90 days.

Patients with the endpoint events of sICH and stent occlusion had a poor clinical outcome: Of the total of 15 patients with sICH, 10 died during the hospital stay, the remaining 5 were discharged with an mRS of 5, with 2 of these patients dying within the next 3 months. From the 40 patients with hemorrhage described by image morphology without relevant neurological deterioration (<4 points in the NIHSS), 12 (30%) showed a good outcome with an mRS ≤2 at discharge. In this group, no patient died until discharge. After 90 days, 15 patients (37.5%) in this group showed an mRS ≤2.

Of the 18 patients with stent occlusion, 3 died, 11 were discharged with an mRS ≥3, 4 (22%) showed a good outcome with mRS ≤2 after 90 days. DAPT use during hospitalization showed even a trend toward a reduction in sICH (*p* = 0.026) no longer significant after Bonferroni-correction.

## 4 Discussion

Our study aimed to analyze the spectrum of APT regimens used in high-volume centers within the GSR for patients with tandem lesions undergoing acute ET with thrombectomy and CAS, focusing on their impact on clinical outcomes and complication rates. The results revealed considerable variability in APT strategies across centers, emphasizing the lack of standardized protocols. A key finding of our comprehensive analysis was that both the type and timing of APT initiation significantly influenced patient outcomes. The use of DAPT within the first day after thrombectomy and CAS in tandem lesions led to better clinical outcome. Specifically, early DAPT was linked to a reduced rate of occlusive stent thrombosis without an increased risk of sICH. To date, no data has addressed the optimal timing of DAPT initiation, highlighting the novelty and potential clinical relevance of these findings.

Our subgroup analysis of GSR data in this patient cohort confirmed findings from previous studies, particularly by further supporting that effective DAPT reduces the rate of poor clinical outcomes in this specific patient cohort. This effect may be explained by the prevention of acute or subacute stent thrombosis, a benefit also reported in the literature (4). However, aggressive DAPT was not shown to be significantly beneficial in the periinterventional phase. Variations in periprocedural management, such as differences in dosing and type of medication across study sites, may explain this lack of effect. Similarly, a recent meta-analysis also found no significant influence of periinterventional APT (13). In particular, prior research has already shown that antithrombotic therapy with antiplatelets agents is not a significant risk factor for sICH or poor clinical outcome (mRS > 2) (5, 6, 14, 15). On the contrary, our analysis found that patient who did not receive aggressive DAPT had a higher incidence of both sICH and asymptomatic ICH. In line with other studies, asymptomatic ICH was also not significantly associated with poor clinical outcomes (5, 16). Additionally, aggressive peri- and postinterventional DAPT has not been associated with an increased rate of symptomatic bleeding complications (16). On the contrary, our analysis found that patient who did not receive in the group without aggressive antiplatelet therapy APT had a higher incidence, there were even more cases of both sICH and ICH. However, in line with other studies, asymptomatic ICH was not significantly associated with poor clinical outcomes (5, 16). In our study effective Our study adds new evidence by demonstrating that effective double antiplatelet therapy DAPT reduced the rate of poor clinical outcomes in this specific patient cohort, which. This may be explained by a significant effect on the prevention of acute or subacute stent thrombosis, a benefit also reported which is also described in the literature (4). Even the risk of sICH showed a trend towards a reduction with antiplatelet therapy APT. This could be explained by its role in preventing ischemic vascular damage, as well as by the fact that high-risk patients with evidence of space-occupying hemorrhage on cCT the following day did not receive further DAPT. The general benefit of APT in these patients is hereby reaffirmed, as it is associated with better clinical outcomes and not with increased rates of sICH, in line with the majority of other studies and recent registry data from larger cohorts (3, 5, 14, 15, 17–19). Given these converging lines of evidence, it seems questionable whether it is still justified to randomize patients between angioplasty and CAS to avoid aggressive DAPT (13).

In our study, the rates of sICH at 6.7% and stent occlusion at 8.1% align with the spectrum of outcomes reported in existing literature (7, 15). Moreover, the rate of good clinical outcomes after 90 days was 36.8%, which, although at the lower end, remains within the range published for patients with tandem lesions (19, 20). Note that the patient sample represented in the GSR reflects real-world data, as patient selection did not always adhere to the study protocols, and included patients likely to have a poor outcome, particularly those receiving rescue treatment. However, the outcomes for this subgroup of patients with tandem lesions are comparable to the previously published results for the entire GSR-study population (10). Additionally, suboptimal APT regimes may contribute to higher rates of stent thrombosis and occlusion (4). Our findings also reaffirm that IVT or the use of aggressive antiplatelets agents, such as Tirofiban or Ticagrelor, does not significantly increase bleeding risks, aligning with other studies (5, 8, 21). Due to the low number of patients receiving antiplatelet agents other than ASA or Clopidogrel, their effect on periinterventional hemorrhages may be underestimated. Further studies could contribute to a better estimation of their risks and benefits (9).

Several important factors known to contribute to poor functional outcome and increased mortality rates among stroke patients with tandem lesions were reaffirmed in this study. These potential confounders include severe neurological impairment (NIHSS scores >15) and infarct load (ASPECTS <6) at admission, incomplete intracranial recanalization (mTICI 0-2a), advanced age (above 60 years), and presence of atrial fibrillation (22, 23).

## 5 Limitations

Our retrospective analysis of registry data and corresponding clinical records has several limitations. Despite a relatively large cohort of 223 cases with tandem lesions, the low rates of endpoint events in subgroups with different antithrombotic regimen may limit the generalizability of our statistical results. The complex analysis required and the extensive cooperation of the participating centers meant that our study concluded in January 2020. Since then, the GSR has expanded, and it would be interesting to investigate whether therapeutic standards and outcomes have improved. Like other studies, our analysis does not provide sufficient data to determine which type and dose of antiplatelet agents are the best options for establishing an effective and safe platelet inhibition in an emergency setting, whether GPIIb-IIIa-inhibitors like Tirofiban or ASA, or a loading dose of Clopidogrel or Ticagrelor. Most centers preferred a combination of ASA and Clopidogrel and often delayed the onset of DAP beyond the first 12–24 h. Despite selecting experienced centers for this analysis, we observed large variations in practice and a lack of standardization across the GSR. Given these variations, which may be driven by personal preferences, further RCT may be necessary to assess more or less aggressive and timed approaches to DAPT.

## 6 Conclusion

Our study demonstrates that initiating APT, particularly DAPT, at least on the first postprocedural day significantly improves clinical outcomes in patients undergoing thrombectomy and CAS for the ET of tandem lesions. This strategy was associated with a reduced risk of stent occlusion and did not increase the rates of sICH. These findings underscore the importance of timely antithrombotic therapy in this patient cohort.

## German Stroke Registry - Endovascular Treatment (GSR-ET) – Steering Committee

Joachim Röther (Asklepios Klinik Altona, Hamburg), Bernd Eckert (Asklepios Klinik Altona, Hamburg), Michael Braun (Bezirkskrankenhaus Günzburg), Gerhard F. Hamann (Bezirkskrankenhaus Günzburg), Eberhard Siebert (Charité – Campus Benjamin Franklin und Mitte, Berlin); Christian H Nolte (Charité – Campus Benjamin Franklin und Mitte, Berlin), Martina Petersen (Klinikum Osnabrück), Lars Krause (Klinikum Osnabrück), Tobias Boeckh-Behrens (Klinikum r.d.Isar, München), Silke Wunderlich (Klinikum r.d.Isar, München), Maximilian Schell (UKE Hamburg-Eppendorf), Jens Fiehler (UKE Hamburg-Eppendorf), Götz Thomalla (UKE Hamburg-Eppendorf), Fabian Flottmann (UKE Hamburg-Eppendorf), Anna Alegiani (Asklepios Klinik Altona, Hamburg), Franziska Dorn (Universitätsklinikum Bonn), Gabor Petzold (Universitätsklinikum Bonn), Jan Hendrik Schäfer (Universitätsklinikum Frankfurt am Main), Fee Keil (Universitätsklinikum Frankfurt am Main), Hanna Zimmermann (Universitätsklinikum München, LMU), Steffen Tiedt (Universitätsklinikum München, LMU), Lars Kellert (Universitätsklinikum München, LMU), Ulrike Ernemann (Universitätsklinik Tübingen), Sven Poli (Universitätsklinik Tübingen), Arno Reich (Uniklinik Aachen (RWTH)), Omid Nikoubashman (Universitätsklinik Aachen (RWTH)), Ilko L Maier (Universitätsklinik Göttingen), Marielle S Ernst (Universitätsklinik Göttingen), Mario Abruscato (Klinikum Hanau), Sven Thonke (Klinikum Hanau), Arman Gregor (Klinikum Nordstadt, Hannover), Evdokia Evangelidou (Klinikum Nordstadt, Hannover), Peter Schellinger (Johannes Wesling Klinikum Minden), Jan Borggrefe (Johannes Wesling Klinikum Minden).

## Data Availability

The data analyzed in this study is subject to the following licenses/restrictions: The data that support the findings of this study are available from the German-stroke-registry (GSR) but restrictions apply to the availability of these data, which were used under license for the current study, and so are not publicly available. Data are however available from the authors upon reasonable request and with permission of the GSR-Steering Committee and the consent of the responsible data officer of the relevant clinics. Requests to access these datasets should be directed to GSR German Stroke Registry Data and Site Managementmaximilian.schell@uke.de.
